# Novel CT radiomics models for the postoperative prediction of early recurrence of resectable pancreatic adenocarcinoma: A single‐center retrospective study in China

**DOI:** 10.1002/acm2.70092

**Published:** 2025-04-11

**Authors:** Xinze Du, Yongsu Ma, Kexin Wang, Xiejian Zhong, Jianxin Wang, Xiaodong Tian, Xiaoying Wang, Yinmo Yang

**Affiliations:** ^1^ Department of Hepatobiliary and Pancreatic Surgery Peking University First Hospital Beijing China; ^2^ Department of Radiology Peking University First Hospital Beijing China

**Keywords:** computed tomography, deep learning, early recurrence, Pancreatic adenocarcinoma, radiomics

## Abstract

**Purpose:**

To assess the predictive capability of CT radiomics features for early recurrence (ER) of pancreatic ductal adenocarcinoma (PDAC).

**Methods:**

Postoperative PDAC patients were retrospectively selected, all of whom had undergone preoperative CT imaging and surgery. Both patients with resectable or borderline‐resectable pancreatic cancer met the eligibility criteria in this study. However, owing to the differences in treatment strategies and such, this research mainly focused on patients with resectable pancreatic cancer. All patients were subject to follow‐up assessments for a minimum of 9 months. A total of 250 cases meeting the inclusion criteria were included. A clinical model, a conventional radiomics model, and a deep‐radiomics model were constructed for ER prediction (defined as occurring within 9 months) in the training set. A model based on the TNM staging was utilized as a baseline for comparison. Assessment of the models' performance was based on the area under the receiver operating characteristic curve (AUC). Additionally, precision‐recall (PR) analysis and calibration assessments were conducted for model evaluation. Furthermore, the clinical utility of the models was evaluated through decision curve analysis (DCA), net reclassification improvement (NRI), and improvement of reclassification index (IRI).

**Results:**

In the test set, the AUC values for ER prediction were as follows: TNM staging, ROC‐AUC = 0.673 (95% CI: 0.550, 0.795), PR‐AUC = 0.362 (95% CI: 0.493, 0.710); clinical model, ROC‐AUC = 0.640 (95% CI: 0.504, 0.775), PR‐AUC = 0.481 (95% CI: 0.520, 0.735); radiomics model, ROC‐AUC = 0.722 (95% CI: 0.604, 0.839), PR‐AUC = 0.575 (95% CI: 0.466, 0.686); and deep‐radiomics model, which exhibited the highest ROC‐AUC of 0.895 (95% CI: 0.820, 0.970), PR‐AUC = 0.834 (95% CI: 0.767, 0.923). The difference in both ROC‐AUC and PR‐AUC for the deep‐radiomics model was statistically significant when compared to the other scores (all *p* < 0.05). The DCA curve of the deep‐radiomics model outperformed the other models. NRI and IRI analyses demonstrated that the deep‐radiomics model significantly enhances risk classification compared to the other prediction methods (all *p* < 0.05).

**Conclusion:**

The predictive performance of deep features based on CT images exhibits favorable outcomes in predicting early recurrence.

## 1 INTRODUCTION

Pancreatic cancer currently exhibits one of the worst prognosis among all solid tumors, with a progressively rising incidence and mortality rate over time.^1^ Surgical intervention stands as the optimal approach to enhance prognosis in pancreatic cancer patients and remains the primary choice for managing resectable cases.^2^ However, due to an absence of effective early diagnostic and screening methods for pancreatic cancer, 80% of patients are diagnosed at advanced or borderline resectable stages, thereby missing out on or delaying their opportunity for surgical treatment.^3^ Even within the relatively fortunate 20% who qualify for surgery, there exist limited advancements in prognosis. Moreover, some patients encounter recurrence within 1 year or even within 6 months post‐surgery and succumb within the subsequent 2–6 months.^4^ Some studies propose that this may be attributed to micro‐metastases and local progression that elude identification by radiologist before the surgery.^5^ The heterogeneity observed in postoperative recurrence rates and survival outcomes among pancreatic cancer patients underscores not only the need to differentiate them based on criteria such as resectable, borderline resectable or unresectable but also necessitates more pertinent approaches to distinguish them according to prognosis so as to guide a more individualized preoperative and postoperative treatments.

Early recurrence (ER) of pancreatic cancer is a novel concept that has recently garnered significant attention in the medical community.^6^ Prominent hospitals and institutions worldwide have defined this phenomenon through diverse clinical studies. However, due to different sample sizes and regional differences in each study, different results were obtained when defined the ER as within 6 months, 9 months, and 12 months.^7^, ^8^, ^9^, ^10^ A recent multicenter retrospective study utilizing data from the China Pancreas Data Center (CPDC), encompassing a substantial sample size of over 3000 patients, unequivocally identified ER within 9 months as the most prognostically significant timeframe for Chinese patients.^11^ Subsequent investigations have endeavored to predict ER using clinical parameters such as CA19‐9 levels, CTCs, MRI, and CT imaging; nevertheless, these studies are limited by small sample sizes, lack of precise definition for ER, and suboptimal predictive efficacy.^12^, ^13^, ^14^ Consequently, there remains an unmet need for region‐specific predictive models tailored specifically towards postoperative ER. The development of more accurate predictive models holds promise in guiding precision medicine approaches for individualized management of pancreatic cancer patients.

CT, particularly enhanced CT, currently serves as the primary modality for screening and diagnosing pancreatic cancer.^15^ It is also routinely employed as the initial imaging examination in nearly all patients with pancreatic cancer during diagnosis and treatment due to its rapid and standardized imaging approach that is widely applicable and cost‐effective.^16^ In this study, we focused on patients with resectable pancreatic cancer and utilized their enhanced CT scans as the primary data source for collection and analysis to construct a predictive model. Radiomics, an emerging discipline with significant potential applications, involves gathering and analyzing comprehensive features and data from medical images to predict disease diagnosis, prognosis analysis, pathological characteristics, and drug treatment effects.^17^, ^18^, ^19^, ^20^, ^21^ The mining and analysis of such data do not necessitate additional invasive procedures, making it convenient in clinical practice while reducing patient discomfort, financial burden, and psychological stress.

The objective of this study is to employ radiomics methods based on the definition of ER within 9 months after radical resection provided by CPDC database. By integrating preoperative clinical data along with preoperative CT data from patients undergoing surgery for pancreatic cancer, our aim is to develop a model that predicts early postoperative recurrence or metastasis of pancreatic cancer. This will facilitate individualized precision medicine for patients without introducing extra costs or invasive procedures.

## 2 MATERIAL AND METHODS

### 2.1 Data collection

Clinical data pertaining to patients undergoing treatment for PDAC were sourced from our hospital's medical records. The data compilation included: demographic details such as gender and age; pre‐surgical metrics like body mass index (BMI), CA19‐9 levels, and tumor diameter measured on CT images; intra‐operative details encompassing surgical methods and superior mesenteric artery (SMA) and vein invasion status; and post‐operative pathology reports detailing peritoneal and lymph node metastasis, diagnosis, tumor differentiation, and pTNM staging. Additionally, post‐operative complications, notably pancreatic fistula (PF), and their treatment approaches were documented. Preoperative CT imaging data were also gathered.

**FIGURE 1:**
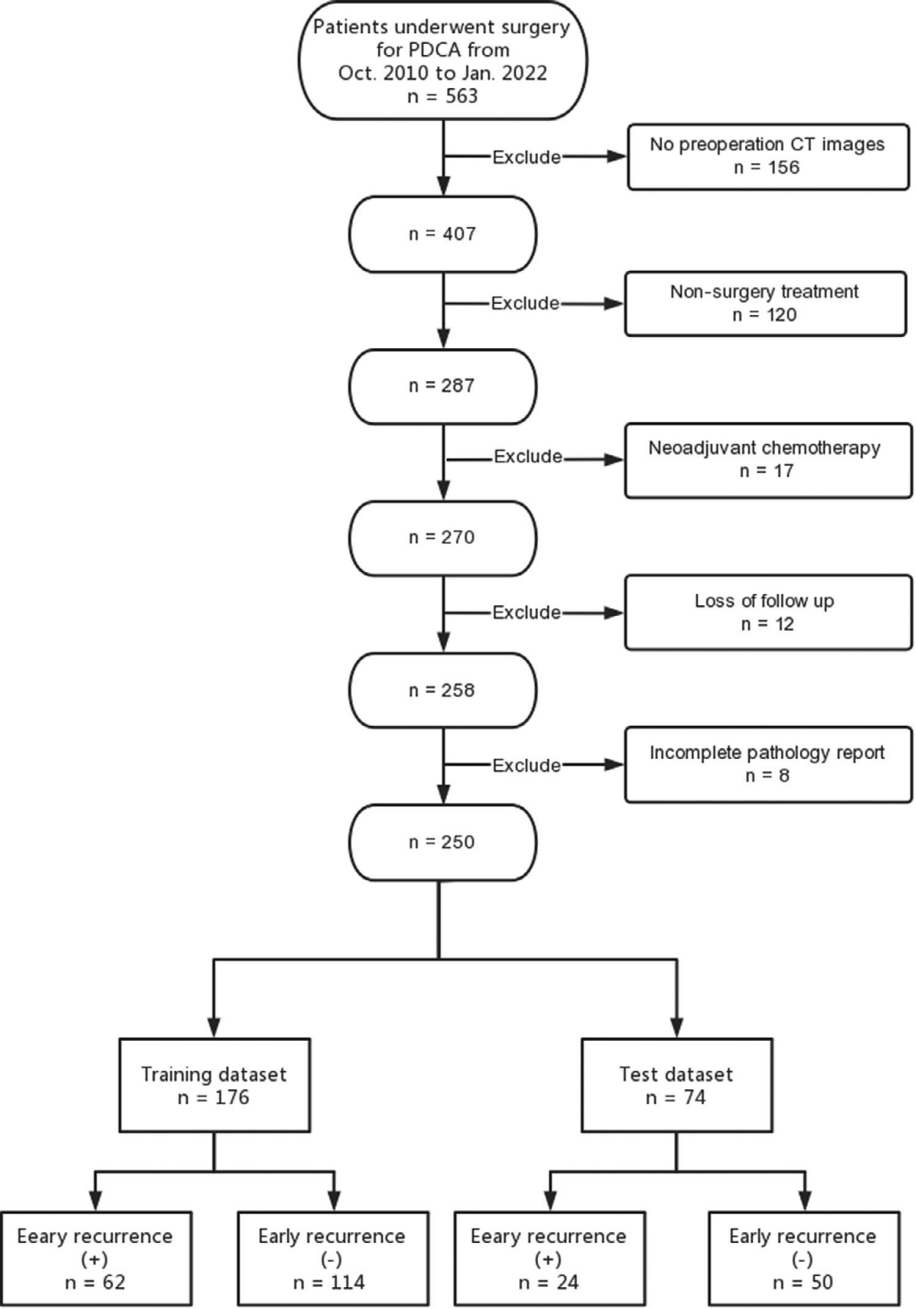
Data enrollment. Patients who underwent surgery for PDAC were enrolled. After exclusion of patients with no of pre‐operative CT images (*n* = 156), non‐surgical treatment (*n* = 120), concurrent neoadjuvant chemotherapy (*n* = 17), loss of follow‐up (*n* = 12), and incomplete pathology reports (*n* = 8), a total of 250 patients were included in the study. The included cohort was randomly split into a training dataset (*n* = 176) and a test dataset (*n* = 74).

Inclusion in the study was limited to patients with a confirmed PDAC diagnosis. Exclusion criteria eliminated patients lacking pre‐operative CT scans, those receiving non‐surgical treatments, undergoing concurrent neoadjuvant chemotherapy, lost to follow‐up, or with incomplete pathology reports. This process resulted in a study cohort of 250 eligible patients, as depicted in Figure 1.

Study participants were monitored for a minimum of 9 months. The term “early recurrence” was specifically defined for this research as the occurrence of a recurrence event within the first 9 months post‐surgery.

### 2.2 CT scanning techniques

Preoperative contrast‐enhanced CT scans were conducted using seven scanners from four different manufacturers. The images had a consistent slice thickness of 1.25 mm and a slice spacing of 5.00 mm, with pixel spacing ranging from 0.69 to 0.77 mm, averaging at 0.73 mm. For a comprehensive depiction of the image acquisition parameters, refer to Table 1.

**TABLE 1:** Image acquisition protocols of the contrast‐enhanced CT.

	GE	NMS	PHILIPS	SIEMENS	Overall
	(*N* = 183)	(*N* = 1)	(*N* = 43)	(*N* = 23)	(N = 250)
Model name					
Brilliance 64	0 (0%)	0 (0%)	17 (39.5%)	0 (0%)	17 (6.8%)
Discovery CT750 HD	159 (86.9%)	0 (0%)	0 (0%)	0 (0%)	159 (63.6%)
iCT 256	0 (0%)	0 (0%)	26 (60.5%)	0 (0%)	26 (10.4%)
LightSpeed VCT	23 (12.6%)	0 (0%)	0 (0%)	0 (0%)	23 (9.2%)
LightSpeed 16	1 (0.5%)	0 (0%)	0 (0%)	0 (0%)	1 (0.4%)
NeuViz Prime	0 (0%)	1 (100%)	0 (0%)	0 (0%)	1 (0.4%)
Somatom Definition Flash	0 (0%)	0 (0%)	0 (0%)	23 (100%)	23 (9.2%)
Slice thickness (mm)					
	1.25 [1.25,1.25]	1.00 [1.00,1.00]	1.00 [1.00,1.00]	1.00 [1.00,1.00]	1.25 [1.25,1.25]
Slice spacing (mm)					
	5.00 [5.00,5.00]	1.00 [1.00,1.00]	1.00 [−1.00,1.00]	5.00 [5.00,5.00]	5.00 [5.00,5.00]
Reconstruction diameter (mm)					
	368 [350,390]	384 [384,384]	383 [364,403]	381 [367,395]	374 [352,392]
Pixel spacing (mm)					
	0.719 [0.684,0.761]	0.750 [0.750,0.750]	0.748 [0.710,0.787]	0.744 [0.717,0.771]	0.730 [0.687,0.766]

Continuous values are median [IQR], and categorical values are number of patients (%).

### 2.3 Region of interest identification

**FIGURE 2:**
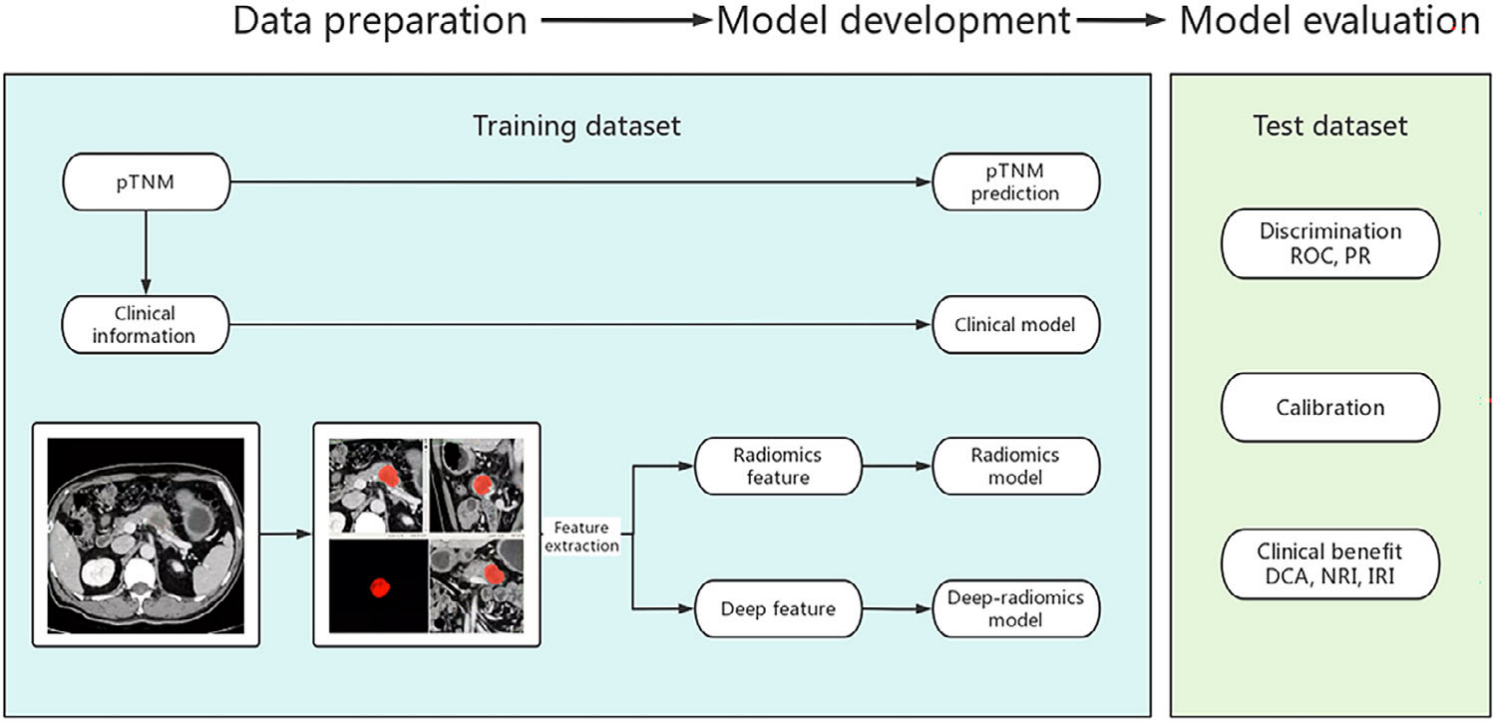
Model development and evaluation. The cases were randomly divided into a training set and a test set. pTNM prediction, a clinical model, a radiomics model, and a deep‐radiomics model were developed using the training dataset and assessed on the test dataset. ROC and PR analyses were employed to evaluate the models' discrimination ability between the ER (+) and ER (−) cases. The calibration curve was utilized to ensure that the predicted probabilities aligned closely with the actual probabilities. DCA, NRI, and IRI were used to evaluate the clinical benefit of the models gained over pTNM staging.

The initial identification of pancreatic lesion areas was performed by a radiologist in training and subsequently reviewed and refined by an experienced abdominal radiologist to ensure accuracy with the pathological findings. This process was carried out slice by slice throughout the CT images, as illustrated in Figure 2. The refined areas were designated as the ROI, which was used for the subsequent extraction of features using radiomics and deep‐radiomics methodologies.

### 2.4 Feature extraction process

For the construction of a traditional radiomics model, we utilized the PyRadiomics package in Python to extract features from the ROIs on CT images. This package is a comprehensive tool designed to standardize the radiomic feature extraction process. To mitigate the impact of scanner variability, image normalization was implemented to reduce confounding factors. The normalization process involved the following steps:

1. Intensity Rescaling: We rescaled the signal intensity to a fixed range (0–65535) across all images to standardize scanner calibration differences.
2. Feature‐wise Normalization: Feature normalization was performed using z‐scores derived from the training dataset's mean and SD, which were subsequently applied to the test dataset to avoid information leakage.

The ROIs were adjusted to a standard size to ensure consistency. The extraction process yielded a comprehensive set of 112 features, including 14 shape features, 18 first‐order statistical features, and 70 texture features. For an in‐depth understanding of the mathematical formulas and the semantic meaning behind these features, refer to the PyRadiomics documentation.

In the realm of deep‐radiomics, a deep learning approach was employed for feature extraction. This method involved several key stages:

1. CT Image Preprocessing: The process began with normalizing the image intensities to achieve uniformity.
2. ROI Resampling: To ensure a uniform voxel size across all ROIs, resampling was performed. The resampling process can be detailed as follows:
  a). Determine Current Pixel Spacing: We first ascertained the original pixel spacing of the CT images, which is provided in the image metadata. This spacing defines the physical size of each voxel in the image data.
  b). Calculate Resize Factor: We then calculated the resize factor by dividing the original spacing by the desired new spacing. In our case, the target was to achieve a uniform voxel size of 1 × 1 × 1 mm^3^ to simplify further processing and analysis.
  c). Resizing the Image: Using the calculated resize factor, we resized the image using interpolation methods provided by image processing libraries (scipy.ndimage). The zoom function was utilized to scale the image according to the resize factor. This function applies interpolation to calculate new pixel values based on the surrounding pixels, which helps in maintaining the image features while changing the resolution.
  d). Interpolation: We used cubic interpolation (order = 3) for resampling, which provides a good balance between smoothness and preservation of image details. This method is particularly effective for medical images where fine details need to be preserved.
  e). Output: The resampled image was then used for further analysis, ensuring that all ROIs were analyzed at the same voxel resolution, which is essential for accurate and reproducible results.
3. Deep Feature Extraction: A pre‐trained ResNet‐50 deep learning model was applied to extract features from the segmented ROIs. This process leveraged the model's learned representations by feeding the ROIs into the convolutional layers of the MedicalNet architecture, which was pre‐loaded with pre‐trained weights, facilitating the extraction of distinctive features. The ResNet‐50 model consists of 50 layers, including convolutional layers, batch normalization layers, and shortcut connections (residual connections). The network starts with an initial 7 × 7 convolutional layer followed by max pooling. It then has four blocks of residual units, with the number of layers in each block being 3, 4, 6, and 3, respectively. The first convolutional layer has 64 filters, and the number of filters increases (to 128, 256, 512) as the network goes deeper. The initial 7 × 7 convolution uses a stride of 2 and padding of 3. The 3 × 3 convolutions in the residual units generally use a stride of 1 and padding of 1. Each convolutional layer is followed by batch normalization to stabilize learning and accelerate convergence. The activation function used throughout the network is the Rectified Linear Unit (ReLU). Each residual unit has a skip connection that adds the input to the output of the residual unit, helping to mitigate the vanishing gradient problem in deep networks. For a comprehensive description of the network, including the specific weights and other hyperparameters, we refer to the original work and the MedicalNet GitHub repository, which provides detailed documentation and code (https://github.com/Tencent/MedicalNet).
4. Feature Dimension Reduction: The channel feature maps produced were condensed through a dimension reduction technique that filtered the maps to retain the maximum values, culminating in a concise set of 512 one‐dimensional features.

### 2.5 Model development and assessment

In our study, we adopted a systematic approach to dataset partitioning to ensure both randomness and representativeness. Specifically, we used a random grouping method with a fixed seed (seed = 123) to ensure reproducibility, and we maintained a partition ratio of 7:3 for the training and test datasets, respectively.

The training set was used to develop three distinct models, which were then evaluated using the test set.

1. Clinical Model Construction: The clinical model was formulated by incorporating the clinical variables such as gender, age, BMI, CA19‐9 levels, tumor diameter, surgical procedure types, SMA and vein invasion presence, peritoneal and lymph node metastasis occurrence, pathology diagnosis, tumor differentiation, pTNM staging, post‐operative PF occurrence, and treatment methods. Univariate logistic regression was initially applied, followed by forward stepwise regression to refine the model, prioritizing the reduction of the Akaike information criterion (AIC). The resulting model's predictors were reported with their odds ratios and confidence intervals (CI).
2. Radiomics and Deep‐Radiomics Model Construction: For the radiomics and deep‐radiomics models, a *z*‐score normalization process was conducted to standardize the extracted features. Pearson correlation coefficients (PCCs) were calculated to detect and eliminate highly correlated features, removing those with PCC values above 0.99 to mitigate multicollinearity. Recursive feature elimination (RFE) was then used to further reduce feature dimensionality by focusing on the importance of feature weights. The gradient boosting machine (GBM) served as the classifier for these models.
3. Predictive Performance Evaluation: The predictive capabilities of the pTNM staging, clinical, radiomics, and deep‐radiomics models were evaluated using the test dataset. The area under the receiver operating characteristic (ROC) curve (AUC) was the primary metric for assessing accuracy. Precision‐recall (PR) analysis was conducted to highlight the models' ability to correctly identify positive cases, particularly in imbalanced datasets. Calibration analysis ensured that the predicted probabilities closely matched actual outcomes. Decision curve analysis (DCA) provided insights into the clinical relevance and practicality of the models' predictions. Finally, net reclassification improvement (NRI) and improvement of reclassification index (IRI) were calculated to quantify the enhancement in the classification of ER cases by each model in comparison to the others.

### 2.6 Statistical analysis methodology

Our statistical analysis was executed with the R software package, version 4.3.1. For the presentation of continuous data, median values are reported alongside their interquartile ranges (IQR). Categorical data is summarized by frequency counts (n) and their corresponding percentages (%). The analysis of continuous data involved the use of the Mann‐Whitney *U*‐test for comparing variables. In the case of categorical data, comparisons were made using either the chi‐square test or Fisher's exact test, depending on the data distribution. Statistical significance was determined by a two‐tailed *p*‐value that was less than 0.05. The comparison of AUC was facilitated by the DeLong test.

## 3 RESULTS

### 3.1 Demographic and tumor profile overview

The study encompassed a total of 250 subjects with an average age of 64.0 years, falling within an interquartile range of 57.0 to 70.0 years. The gender distribution was fairly balanced, with 104 females and 146 males. The majority of the cases, 229, were identified as ductal adenocarcinoma. Less common were cholangiocarcinoma (periampullary type) with 11 cases and ampullary carcinoma (periampullary type) with 10 cases. The TNM staging distribution included the following: stage IA with 31 cases, stage IB with 73 cases, stage IIA with 31 cases, stage IIB with 81 cases, and stage III with 34 cases. For a comprehensive breakdown, refer to Tables 2 and 3.

**TABLE 2:** Clinical characteristics in the training and test datasets.

	Overall	Training dataset	Test dataset	*p*‐value
	(*N* = 250)	(*N* = 176)	(*N* = 74)	
Age (year)				
	64.0 [57.0,70.0]	64.0 [57.0,70.0]	64.0 [57.5,70.8]	0.642
Gender				
Female	104 (41.6%)	74 (42.0%)	30 (40.5%)	0.936
Male	146 (58.4%)	102 (58.0%)	44 (59.5%)	
BMI (kg/m^2^)				
	23.4 [21.4,25.8]	23.6 [21.6,25.9]	23.2 [20.8,25.2]	0.292
CA19‐9 level (U/mL)				
	161 [53.5,453]	161 [55.0,452]	173 [43.9,485]	0.901
Tumor diameter (cm)				
	3.00 [2.30,4.00]	3.00 [2.38,4.00]	3.00 [2.30,4.58]	0.997
Surgery type				
Duodeno‐pancreatectomy	167 (66.8%)	121 (68.8%)	46 (62.2%)	0.343
Distal pancreatectomy with Splenectomy	81 (32.4%)	53 (30.1%)	28 (37.8%)	
Total pancreatectomy	2 (0.8%)	2 (1.1%)	0 (0%)	
SMA invasion				
No	241 (96.4%)	168 (95.5%)	73 (98.6%)	0.387
Yes	9 (3.6%)	8 (4.5%)	1 (1.4%)	
Venous invasion				
No	231 (92.4%)	162 (92.0%)	69 (93.2%)	0.948
Yes	19 (7.6%)	14 (8.0%)	5 (6.8%)	
Lymph node metastasis				
No	134 (53.6%)	91 (51.7%)	43 (58.1%)	0.431
Yes	116 (46.4%)	85 (48.3%)	31 (41.9%)	
Peritoneal metastasis				
No	236 (94.4%)	167 (94.9%)	69 (93.2%)	0.83
Yes	14 (5.6%)	9 (5.1%)	5 (6.8%)	
Diagnosis				
Ductal adenocarcinoma	229 (91.6%)	163 (92.6%)	66 (89.2%)	0.661
Cholangiocarcinoma—periampullary type	11 (4.4%)	7 (4.0%)	4 (5.4%)	
Ampullary carcinoma—periampullary type	10 (4.0%)	6 (3.4%)	4 (5.4%)	
Differentiation				
Low	68 (27.2%)	45 (25.6%)	23 (31.1%)	0.112
Mid	139 (55.6%)	105 (59.7%)	34 (45.9%)	
High	43 (17.2%)	26 (14.8%)	17 (23.0%)	
pTNM				
Stage IA	31 (12.4%)	19 (10.8%)	12 (16.2%)	0.508
Stage IB	73 (29.2%)	54 (30.7%)	19 (25.7%)	
Stage IIA	31 (12.4%)	19 (10.8%)	12 (16.2%)	
Stage IIB	81 (32.4%)	59 (33.5%)	22 (29.7%)	
Stage III	34 (13.6%)	25 (14.2%)	9 (12.2%)	
Pancreatic fistula				
No PF	95 (38.0%)	71 (40.3%)	24 (32.4%)	0.506
Grade A	99 (39.6%)	66 (37.5%)	33 (44.6%)	
Grade B	52 (20.8%)	37 (21.0%)	15 (20.3%)	
Grade C	4 (1.6%)	2 (1.1%)	2 (2.7%)	
Treatment PF				
No treatment	232 (92.8%)	166 (94.3%)	66 (89.2%)	0.164
Supportive care and drainage	17 (6.8%)	10 (5.7%)	7 (9.5%)	
Surgical intervention	1 (0.4%)	0 (0%)	1 (1.4%)	

Continuous values are median [IQR], and categorical values are number of patients (%).

**TABLE 3:** Comparison of the clinical characteristics in the recurrence and non‐recurrence groups.

	ER (−)	ER (+)	*p*‐value
	(*N* = 164)	(*N* = 86)	
Age (year)			
	65.0 [59.0,70.0]	62.5 [56.0,70.0]	0.368
Gender			
Female	73 (44.5%)	31 (36.0%)	0.248
Male	91 (55.5%)	55 (64.0%)	
BMI (kg/m^2^)			
	23.4 [21.0,25.8]	23.5 [21.7,25.8]	0.551
CA19‐9 level (U/mL)			
	139 [42.5,331]	208 [75.2,759]	0.045
Tumor diameter (cm)			
	3.00 [2.20,4.00]	3.50 [2.50,4.50]	0.004
Surgery type			
Duodeno‐pancreatectomy	108 (65.9%)	59 (68.6%)	0.791
Distal pancreatectomy with Splenectomy	55 (33.5%)	26 (30.2%)	
Total pancreatectomy	1 (0.6%)	1 (1.2%)	
SMA invasion			
No	158 (96.3%)	83 (96.5%)	>0.999
Yes	6 (3.7%)	3 (3.5%)	
Venous invasion			
No	158 (96.3%)	73 (84.9%)	0.003
Yes	6 (3.7%)	13 (15.1%)	
Lymph node metastasis			
No	104 (63.4%)	30 (34.9%)	<0.001
Yes	60 (36.6%)	56 (65.1%)	
Peritoneal metastasis			
No	157 (95.7%)	79 (91.9%)	0.329
Yes	7 (4.3%)	7 (8.1%)	
Diagnosis			
Ductal adenocarcinoma	148 (90.2%)	81 (94.2%)	0.481
Cholangiocarcinoma (periampullary type)	9 (5.5%)	2 (2.3%)	
Ampullary carcinoma (periampullary type)	7 (4.3%)	3 (3.5%)	
Differentiation			
Low	35 (21.3%)	33 (38.4%)	0.016
Mid	98 (59.8%)	41 (47.7%)	
High	31 (18.9%)	12 (14.0%)	
pTNM			
Stage IA	26 (15.9%)	5 (5.8%)	<0.001
Stage IB	58 (35.4%)	15 (17.4%)	
Stage IIA	21 (12.8%)	10 (11.6%)	
Stage IIB	44 (26.8%)	37 (43.0%)	
Stage III	15 (9.1%)	19 (22.1%)	
Pancreatic fistula			
No PF	63 (38.4%)	32 (37.2%)	0.974
Grade A	64 (39.0%)	35 (40.7%)	
Grade B	34 (20.7%)	18 (20.9%)	
Grade C	3 (1.8%)	1 (1.2%)	
Treatment PF			
No treatment	151 (92.1%)	81 (94.2%)	0.244
Supportive care and drainage	13 (7.9%)	4 (4.7%)	
Surgical intervention	0 (0%)	1 (1.2%)	

The clinical data for the training and test datasets were compared, and no significant differences were noted (*p*‐values for all comparisons were greater than 0.05). However, when distinguishing between patients with ER and those without, significant differences were identified in several factors, including CA 19‐9 levels, tumor diameter, venous invasion, lymph node metastasis, tumor differentiation, and pTNM stage (*p*‐values for all were less than 0.05, as detailed in Table 3).

### 3.2 Model fitting outcomes summary

**FIGURE 3:**
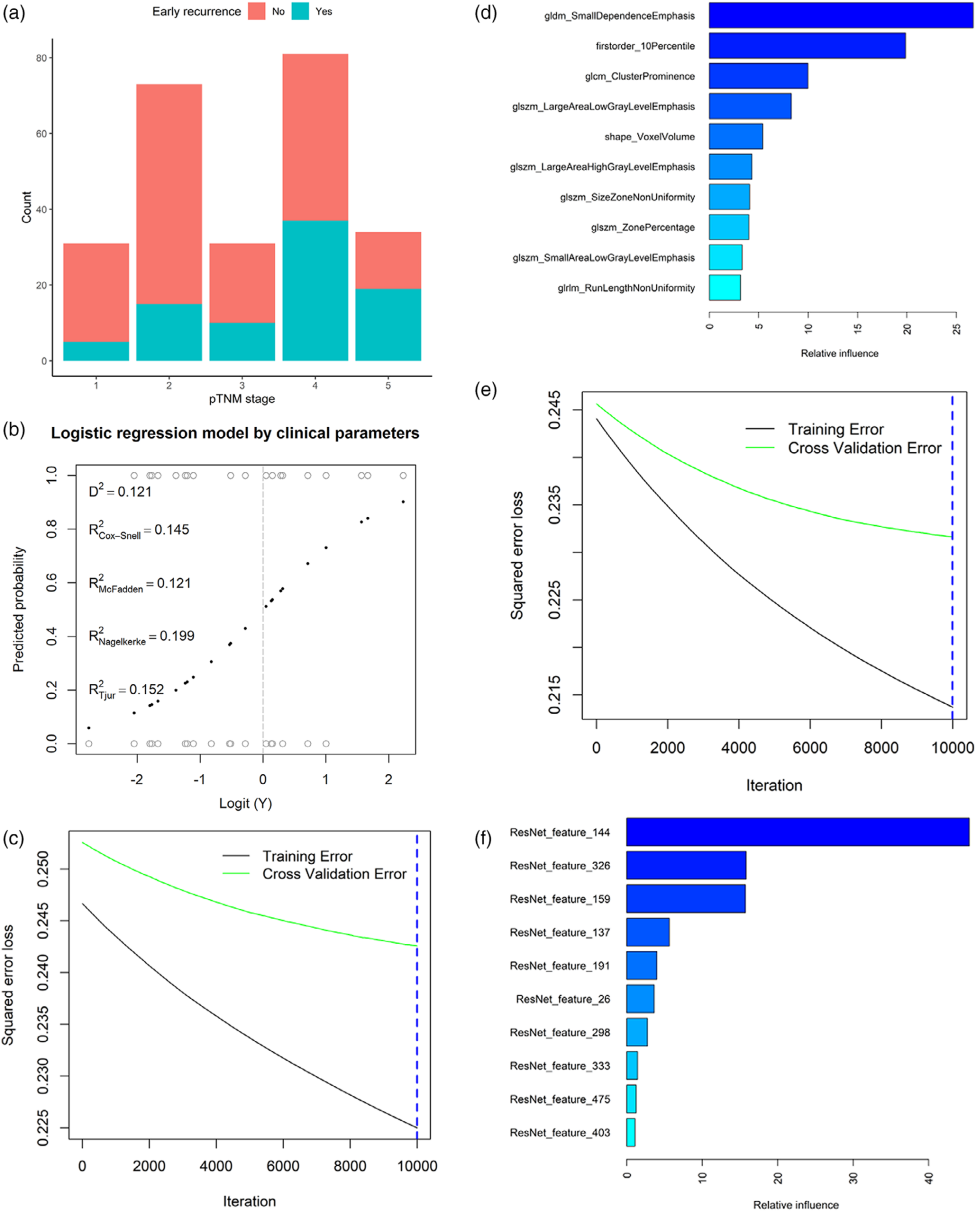
Illustration of the prediction methods. Figure 3a illustrates the occurrence of early recurrence across various TNM stages. In Figure 3b, a logistic regression model is presented, incorporating clinical information. Figure 3c shows the iterative process of gradient boosting applied to the radiomics model. The impact of radiomics features on the model is depicted in Figure 3d. Additionally, Figure 3e demonstrates the iterative process of gradient boosting for the deep‐radiomics model, while Figure 3f illustrates the influence of deep‐radiomics features on the model.

Figure 3a offers a graphical depiction of ER distribution across various pTNM staging groups. The pTNM analysis revealed that patients in Stage III with ER outnumbered those without ER (19:15), while in Stages IA, IB, IIA, and IIB, the number of patients with ER was less than those without recurrence (5:26, 15:58, 10:21, and 37:44, respectively).

The logistic regression model's fitting process is outlined in Figure 3b, with the corresponding odds ratios for both the univariable and multivariable models detailed in Table 4. Univariate logistic regression analysis indicated that venous invasion (OR = 3.70, *p* = 0.025), tumor diameter (OR = 1.24, *p* = 0.039), and tumor differentiation (OR = 0.48, *p* = 0.044) are associated with ER. Multivariate logistic regression analysis further confirmed that venous invasion (OR = 3.77, *p* = 0.038) is associated with ER.

**TABLE 4:** Logistic regression model variables for the classification prediction model by clinical information.

Predictor	Description	ER(‐) (*N* = 114)	ER(+) (*N* = 62)	OR (univariable)	OR (multivariable)
Gender	Female	52 (45.6%)	22 (35.5%)		
	Male	62 (54.4%)	40 (64.5%)	1.52 (0.81–2.88, *p* = 0.195)	1.73 (0.85‐3.51, *p* = 0.131)
Age	Mean ± SD	63.6 ± 9.9	63.9 ± 11.2	1.00 (0.97–1.03, *p* = 0.850)	
BMI	Mean ± SD	23.6 ± 3.1	23.8 ± 2.9	1.03 (0.93–1.14, *p* = 0.615)	
CA19‐9	Mean ± SD	278.1 ± 319.4	360.6 ± 376.6	1.00 (1.00–1.00, *p* = 0.128)	1.00 (1.00–1.00, *p* = 0.937)
Surgery type	Duodeno‐pancreatectomy	76 (66.7%)	45 (72.6%)	NA	
	Distal pancreatectomy with Splenectomy	37 (32.5%)	16 (25.8%)	0.73 (0.37–1.46, *p* = 0.374)	
	Total pancreatectomy	1 (0.9%)	1 (1.6%)	1.69 (0.10–27.67, *p* = 0.713)	
SMA invasion	No	108 (94.7%)	60 (96.8%)		
	Yes	6 (5.3%)	2 (3.2%)	0.60 (0.12–3.07, *p* = 0.539)	
Venous invasion	No	109 (95.6%)	53 (85.5%)		
	Yes	5 (4.4%)	9 (14.5%)	3.70 (1.18–11.59, *p* = 0.025)	3.77 (1.07–13.22, *p* = 0.038)
Pancreatic fistula	No PF	49 (43%)	22 (35.5%)	NA	
	Grade A	39 (34.2%)	27 (43.5%)	1.54 (0.76–3.11, *p* = 0.227)	
	Grade B	24 (21.1%)	13 (21%)	1.21 (0.52–2.80, *p* = 0.662)	
	Grade C	2 (1.8%)	0 (0%)	0.00 (0.00‐Inf, *p* = 0.989)	
Treatment of PF	No treatment	106 (93%)	60 (96.8%)		
	Supportive care and drainage	8 (7%)	2 (3.2%)	0.44 (0.09–2.15, *p* = 0.311)	
Diagnosis	Ductal adenocarcinoma	105 (92.1%)	58 (93.5%)	NA	
	Cholangiocarcinoma (periampullary type)	5 (4.4%)	2 (3.2%)	0.72 (0.14–3.85, *p* = 0.705)	
	Ampullary carcinoma (periampullary type)	4 (3.5%)	2 (3.2%)	0.91 (0.16–5.09, *p* = 0.910)	
Tumor diameter	Mean ± SD	3.2 ± 1.4	3.7 ± 1.8	1.24 (1.01–1.53, *p* = 0.039)	1.12 (0.85–1.48, *p* = 0.429)
pTNM staging	Stage IA	15 (13.2%)	4 (6.5%)		
	Stage IB	43 (37.7%)	11 (17.7%)		
	Stage IIA	14 (12.3%)	5 (8.1%)		
	Stage IIB	32 (28.1%)	27 (43.5%)		
	Stage III	10 (8.8%)	15 (24.2%)		
Differentiation	Low	23 (20.2%)	22 (35.5%)		
	Mid	72 (63.2%)	33 (53.2%)	0.48 (0.23–0.98, *p* = 0.044)	0.53 (0.24–1.15, *p* = 0.106)
	High	19 (16.7%)	7 (11.3%)	0.39 (0.14–1.10, *p* = 0.074)	0.46 (0.14‐1.44, *p* = 0.181)

In the case of the radiomics model, the iterative fitting of the gradient boosting classifier is shown in Figure 3c. The impact of individual features is highlighted in Figure 3d, with an expanded view provided in Table 5. For the deep‐radiomics model, Figure 3e illustrates the classifier's iterative fitting process. The feature influence is further elaborated in Figure 3f, and a more detailed analysis is available in Table 6. As the number of iterations increased, the squared error gradually decreased. The relative importance of the radiomics features ultimately included in the GBM model ranged from 1.173 to 26.622, with gldm_SmallDependenceEmphasis having the highest relative importance.

**TABLE 5:** Relative importance of the features in the radiomics model.

var	rel.inf
gldm_SmallDependenceEmphasis	26.622
firstorder_10Percentile	19.862
glcm_ClusterProminence	9.964
glszm_LargeAreaLowGrayLevelEmphasis	8.306
shape_VoxelVolume	5.329
glszm_LargeAreaHighGrayLevelEmphasis	4.298
glszm_ZonePercentage	4.088
glszm_SizeZoneNonUniformity	4.077
glszm_SmallAreaLowGrayLevelEmphasis	3.301
glrlm_RunLengthNonUniformity	3.236
glszm_LowGrayLevelZoneEmphasis	2.741
glcm_JointEnergy	2.576
gldm_LargeDependenceHighGrayLevelEmphasis	1.708
glcm_Contrast	1.543
glcm_MaximumProbability	1.178
gldm_DependenceVariance	1.173

**TABLE 6:** Relative importance of the features in the deep‐radiomics model.

var	rel.inf
ResNet_feature_144	45.076
ResNet_feature_326	16.159
ResNet_feature_159	16.071
ResNet_feature_137	5.512
ResNet_feature_191	4.065
ResNet_feature_26	3.733
ResNet_feature_298	2.875
ResNet_feature_475	1.283
ResNet_feature_88	1.179
ResNet_feature_333	1.130
ResNet_feature_403	1.073
ResNet_feature_147	0.523
ResNet_feature_2	0.467
ResNet_feature_295	0.430
ResNet_feature_96	0.316
ResNet_feature_50	0.109

Similarly, as the number of iterations increased, the squared error gradually decreased. The relative importance of the deep radiomics features ultimately included in the GBM model ranged from 0.109 to 45.076, with ResNet_feature_144 having the highest relative importance.

### 3.3 Assessment of predictive performance

**FIGURE 4:**
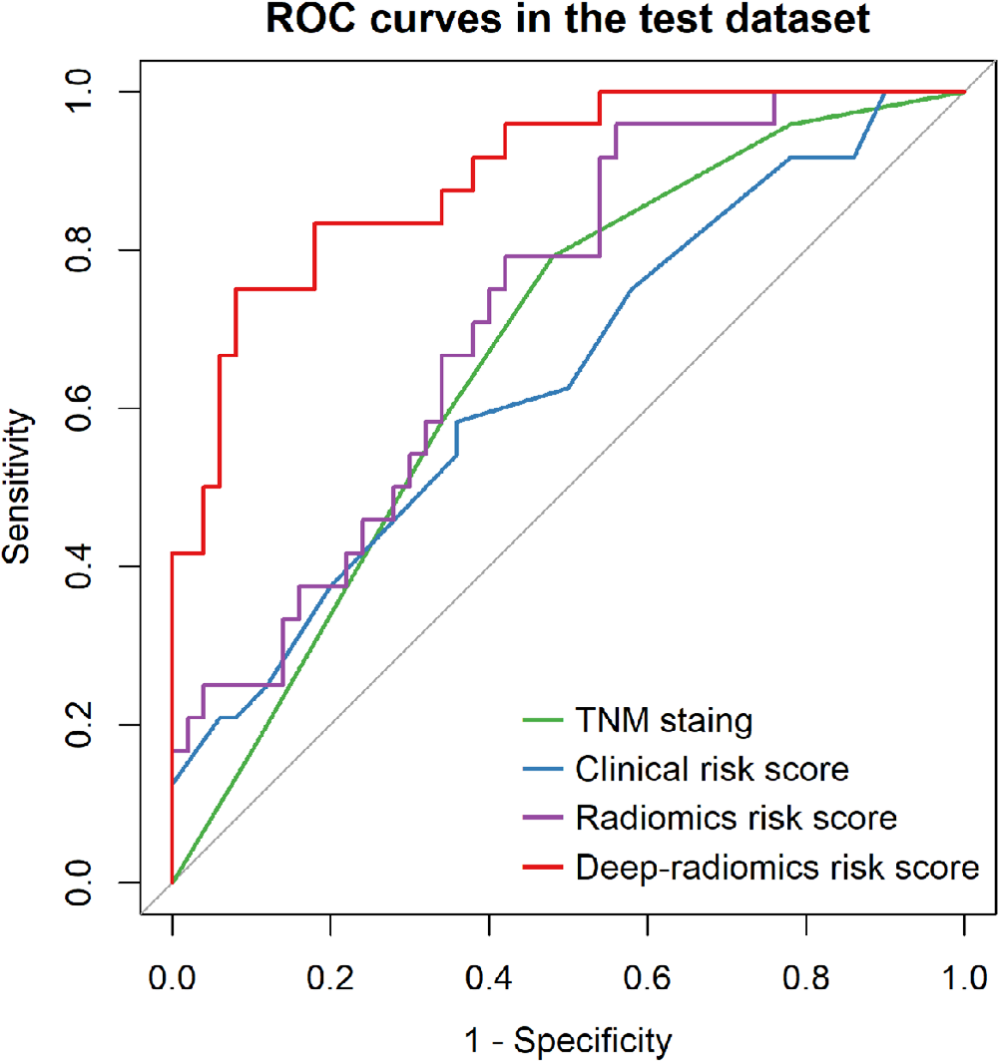
AUCs of the prediction methods. ROC curves of the different prediction methods in the test dataset. The AUC of the deep‐radiomics model significantly outperformed the other scores, with all comparisons showing statistical significance (all *p* < 0.05).

Table 7 presents a detailed summary of predictive performance metrics, encompassing the AUC, accuracy (ACC), sensitivity (SEN), specificity (SPE), positive predictive value (PPV), and negative predictive value (NPV). The TNM staging showed an AUC of 0.673 with a 95% CI from 0.550 to 0.795. The clinical model had an AUC of 0.640 (95% CI: 0.504, 0.775), while the radiomics model achieved an AUC of 0.722 (95% CI: 0.604, 0.839). Notably, the deep‐radiomics model achieved the highest AUC at 0.895 (95% CI: 0.820, 0.970), significantly surpassing the other models in statistical terms (*p* < 0.05). For further insights on AUC comparisons, see Table 8 and Figure 4.

**TABLE 7:** Evaluation of the classification prediction methods in the test dataset.

	pTNM staging	Clinical model	Radiomics model	Deep‐radiomics model
AUCroc	0.673 (0.550, 0.795)	0.640 (0.504, 0.775)	0.722 (0.604, 0.839)	0.895 (0.820, 0.970)
AUCpr	0.362 (0.493, 0.710)	0.481 (0.520, 0.735)	0.575 (0.466, 0.686)	0.834 (0.767, 0.923)
ACC	0.608 (0.602, 0.614)	0.622 (0.615, 0.628)	0.608 (0.602, 0.614)	0.865 (0.862, 0.868)
SEN	0.792 (0.629, 0.954)	0.583 (0.386, 0.781)	0.958 (0.878, 1.000)	0.750 (0.577, 0.923)
SPE	0.520 (0.382, 0.658)	0.640 (0.507, 0.773)	0.440 (0.302, 0.578)	0.920 (0.845, 0.995)
PPV	0.442 (0.293, 0.590)	0.438 (0.266, 0.609)	0.451 (0.314, 0.588)	0.818 (0.657, 0.979)
NPV	0.839 (0.709, 0.968)	0.762 (0.633, 0.891)	0.957 (0.873, 1.040)	0.885 (0.798, 0.971)

**TABLE 8:** Comparison of AUCs in the test dataset.

	TNM staging	Clinical model	Radiomics model	Deep‐radiomics model
TNM staging	NA	0.477	0.474	0.003
Clinical model	0.477	NA	0.291	0.002
Radiomics model	0.474	0.291	NA	0.032
Deep‐radiomics model	0.003	0.002	0.032	NA

**FIGURE 5:**
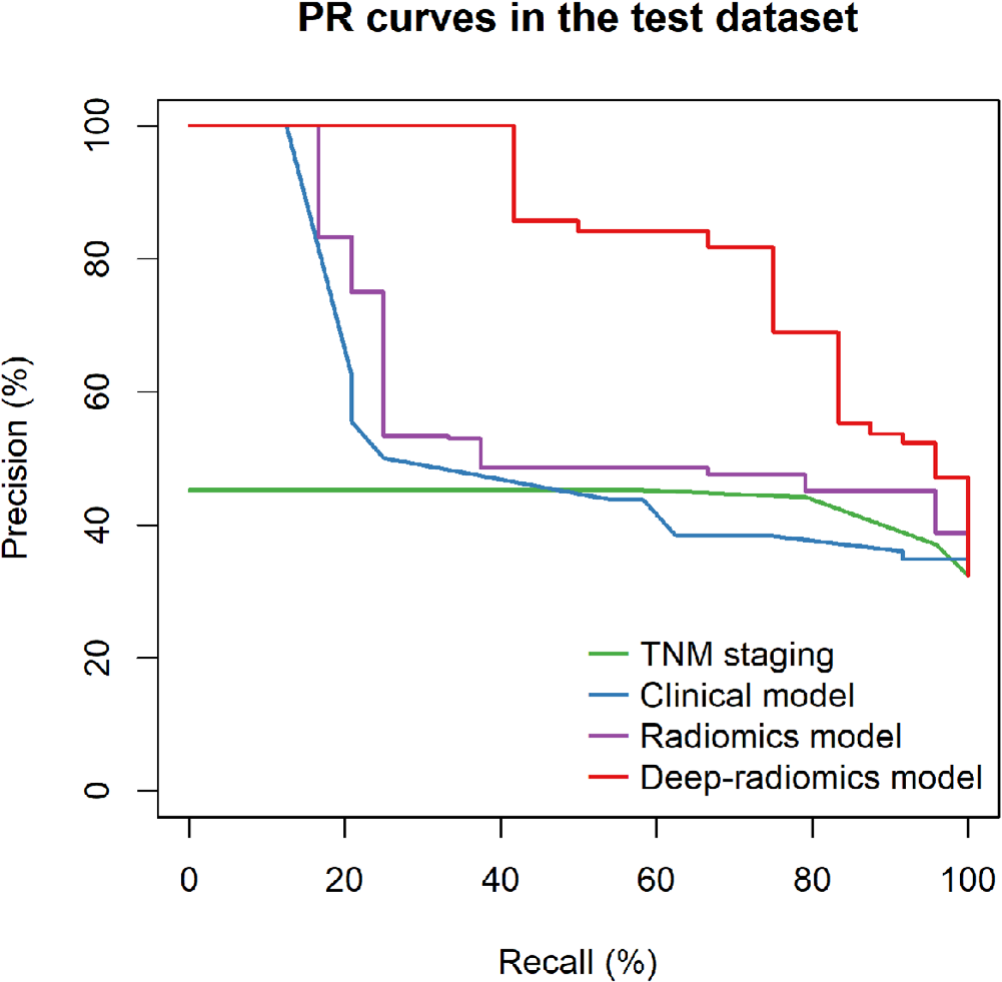
PR curves of the prediction methods. PR curves of the different prediction methods in the test dataset. The confidence intervals around the AUC estimates of the deep‐radiomics model significantly outperformed the other methods.

The AUC for the PR curves were as follows: pTNM staging at 0.362 (95% CI: 0.493, 0.710), the clinical model at 0.481 (95% CI: 0.520, 0.735), the radiomics model at 0.575 (95% CI: 0.466, 0.686), and the deep‐radiomics model at 0.834 (95% CI: 0.767, 0.923). The PR curves, depicted in Figure 5, confirm the statistical significance of the deep‐radiomics model's advantage.

**FIGURE 6:**
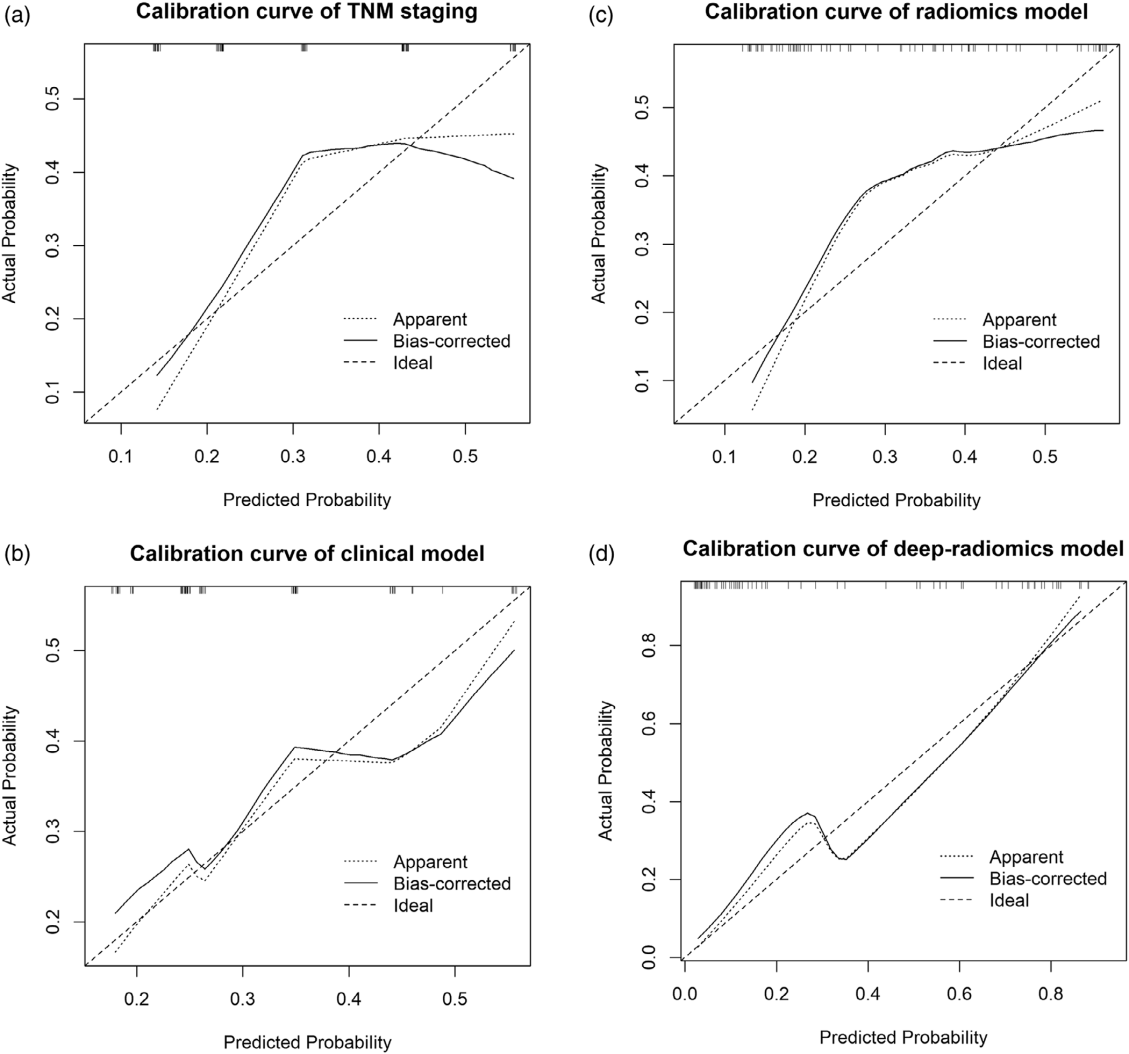
Calibration curves of the prediction methods. Figure 6a to 6d depict the calibration curve for different prediction methods: pTNM staging prediction, clinical model, radiomics model, and deep‐radiomics model.

Figure 6 illustrates the calibration curves for the four predictive methods, with the mean squared error and Brier score for each method indicating their predictive accuracy and reliability. The deep‐radiomics model had the lowest mean squared error and Brier score, suggesting the best alignment between predicted and observed outcomes.

**FIGURE 7:**
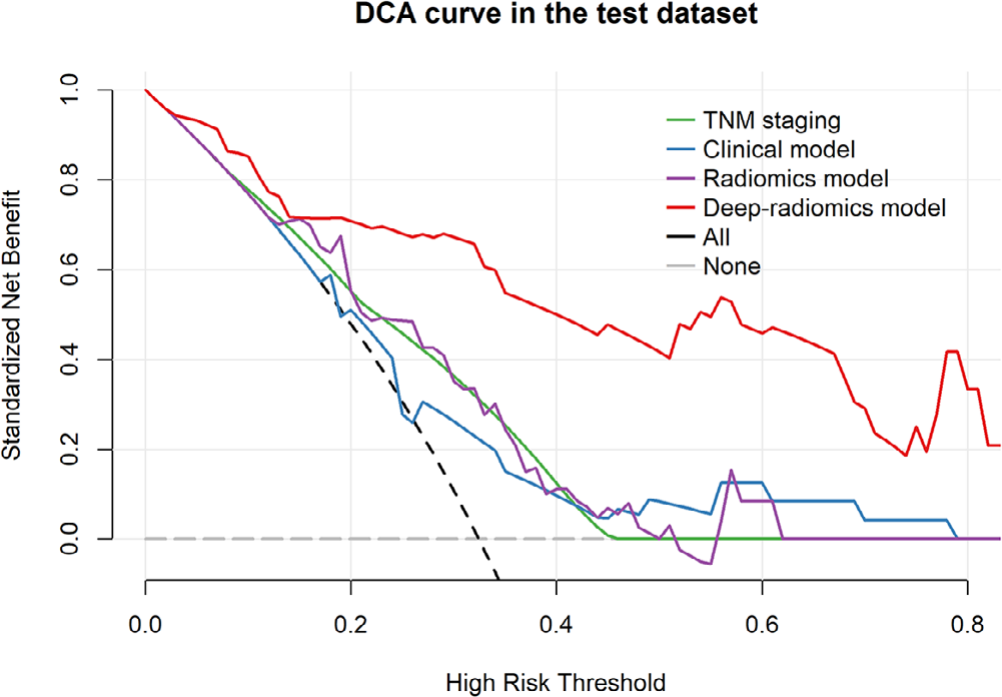
Decision curve analysis of the prediction methods. The DCA curve of the deep‐radiomics model demonstrates the highest clinical utility among the four methods assessed. It provides the greatest net benefit in correctly classifying positive cases while minimizing false positives across a range of 0.4 to 0.8 probability thresholds.

Figure 7 shows the DCA for the prediction methods, highlighting the deep‐radiomics model's superior clinical utility, as it provides the highest net benefit by accurately identifying positive cases while reducing false positives across a range of probability thresholds. The overlapping DCA curves of the other methods suggest similar clinical utility within the specified threshold range.

Table 9 details the NRI and IDI analysis, where the deep‐radiomics model significantly outperformed other methods in terms of reclassification accuracy (*p* < 0.05).

**TABLE 9:** NRI and IRI values in the test dataset.

	TNM staging		Clinical model		Radiomics model		Deep‐radiomics model	
	Value (95% CI)	*p*‐value	Value (95% CI)	*p*‐value	Value (95% CI)	*p*‐value	Value (95% CI)	*p*‐value
**NRI**								
TNM staging	NA	NA	−0.115 (−0.293, 0.063)	0.204	0.083 (−0.267, 0.434)	0.641	0.553 (0.201, 0.905)	0.002
Clinical model	−0.115 (−0.293, 0.063)	0.204	NA	NA	0.355 (−0.033, 0.743)	0.073	0.615 (0.194, 1.037)	0.004
Radiomics model	0.083 (−0.267, 0.434)	0.641	0.355 (−0.033, 0.743)	0.073	NA	NA	0.450 (0.027, 0.873)	0.037
Deep‐radiomics model	0.553 (0.201, 0.905)	0.002	0.615 (0.194, 1.037)	0.004	0.450 (0.027, 0.873)	0.037	NA	NA
**IDI**								
TNM staging	NA	NA	−0.056 (−0.135, 0.023)	0.165	0.043 (−0.088, 0.175)	0.520	0.248 (0.082, 0.414)	0.003
Clinical model	−0.056 (−0.135, 0.023)	0.165	NA	NA	0.099 (−0.034, 0.232)	0.144	0.304 (0.150, 0.458)	<0.001
Radiomics model	0.043 (−0.088, 0.175)	0.520	0.099 (−0.034, 0.232)	0.144	NA	NA	0.205 (0.014, 0.395)	0.035
Deep‐radiomics model	0.248 (0.082, 0.414)	0.003	0.304 (0.150, 0.458)	<0.001	0.205 (0.014, 0.395)	0.035	NA	NA

## 4 DISCUSSION

In the context of the challenging early diagnosis of pancreatic cancer, the opportunity for surgical intervention in patients with this disease is hard‐earned.^22^ When most patients are diagnosed with resectable pancreatic cancer, surgeons typically prioritize surgery as a first‐line treatment option. However, the efficacy of surgical treatment is limited and radical interventions are rare.^23^ Unlike other solid tumors, pancreatic cancer often exhibits rapid recurrence and metastasis following radical surgery, leading to a short survival time after relapse and metastasis.^24^ The commonly used adjuvant therapy based on gemcitabine and 5‐Fu for postoperative treatment of pancreatic cancer has shown suboptimal effectiveness or tolerability.^3^ Consequently, these patients face immediate decisions regarding subsequent adjuvant therapy after undergoing extensive surgical trauma without clear guidance on treatment regimens or cycles. In light of this phenomenon, some scholars have proposed the concept of ER to distinguish between early and late recurrence groups in order to facilitate more precise and individualized treatments. Different studies have yielded varying results due to differences in sample sizes and regional disparities. Based on a multi‐center retrospective data analysis conducted by CPDC, we propose defining 9 months post‐surgery as the time point for distinguishing between ER and late recurrence—a definition that may be most suitable for Chinese patients with pancreatic cancer.

Following the proposal of the concept of ER in pancreatic cancer, numerous studies have attempted to define and predict ER based on their own clinical data.^9^, ^10^, ^14^ However, these studies are constrained by limited sample sizes and other methodological issues, resulting in an inaccurate definition of ER time. Similarly, inadequate model training due to small sample sizes has been observed. Furthermore, some studies have relied on additional invasive procedures to collect clinical specimens for analysis and develop prediction models, which hinders future generalization and fails to provide meaningful guidance for clinical treatment while imposing economic and psychological burdens on patients.^13^, ^25^ In recent years, there have also been imaging‐related articles predicting the prognosis of pancreatic cancer; however, they suffer from similar limitations such as small sample sizes, lack of clinical significance regarding predicted prognosis timeframes, and suboptimal model performance.^26^, ^27^ In this study we successfully developed a prediction model by integrating preoperative enhanced CT images with preoperative and postoperative clinical data to accurately forecast ER and metastasis of resectable pancreatic cancer, surpassing the predictive efficacy of both CT and clinical models. Compared to previous studies based on clinical and imaging data, our model demonstrates superior predictive performance. Moreover, we extend beyond solely examining imaging features within the tumor area delineated by the region of interest (ROI), incorporating relevant CT characteristics of the pancreatic duct and surrounding pancreas outside the tumor boundary, as well as location‐based attributes of the tumor, pancreatic duct, and outer edge of the pancreas. This comprehensive approach enables us to gather and compare imaging‐related data more comprehensively. Overall, our study offers potential guidance for personalized and precise treatment decisions immediately after surgery but prior to initiating postoperative maintenance therapy. To some extent, different postoperative treatment options can be selected based on our model's indications for ER in order to enhance patient prognosis while improving compliance with and tolerance towards adjuvant treatments.

However, our study has certain deficiencies and limitations. Specifically: (1) Currently, we have only conducted internal validation, lacking external validation data; (2) This study is retrospective in nature, which may introduce selection bias; (3) Despite having a relatively large sample size compared to some other studies predicting ER and metastasis, it remains insufficient. Therefore, more data are required to enhance the accuracy of the model; (4) Manual labeling of the ROI for both the pancreas and mass in CT images is prone to errors and requires substantial human resources. Image preprocessing and feature engineering should be systematically evaluated to identify optimal methods in the model development pipeline. Future studies should consider employing automated segmentation techniques and exploring enhanced features to improve efficiency and consistency;(5) Currently, the practical application of prediction models in clinical settings remains limited. Although we have successfully identified ER and metastasis in post‐surgical patients, the subsequent implementation of more personalized treatment strategies has not yet been fully realized. In the future, we aim to actively integrate predictive models into clinical decision‐making processes and rigorously evaluate the tangible benefits these models can provide to patients

Finally, considering the predictive capacity of the radiomics model in ER and metastasis, as demonstrated by current research findings, we will continue to validate and refine our model. Additionally, preliminary prospective studies will be conducted to investigate and confirm the impact of different postoperative treatments on patient prognosis and quality of life based on their classification according to ER using our model. In addition, we will collect patient data, including prognostic information and postoperative quality of life metrics, to evaluate the effectiveness of the radiomics model in guiding postoperative treatment for patients with resectable pancreatic cancer. This represents a significant advancement over the current standardized treatment decision‐making approach.

## 5 CONCLUSION

In this retrospective study, we developed a clinical‐radiomics model based on CT and clinical data of patients, which demonstrated good predictive efficacy. This model can provide surgeons with the probability of early postoperative recurrence for reference immediately after surgery, thus providing a clearer prognosis basis for individualized treatment of pancreatic cancer patients. Furthermore, it has great potential in improving patient prognosis and assisting surgeons in selecting a more appropriate chemotherapy regimen which can achieve a more optimal balance between the efficacy of chemotherapy and its associated toxicity. We will continue to verify and optimize our model through preliminary prospective studies to explore the degree of improvement in different postoperative treatment methods for patients classified according to ER with regards to their prognosis and quality of life.

## AUTHOR CONTRIBUTIONS

*Project development*: Xinze Du, Xiaoying Wang, Yongsu Ma Yang and Yongsu Ma. *Data collection*: Xinze Du, Xiejian Zhong, AH Yan and YP Yan. *Data analysis*: Kexin Wang, Xiaoying Wang and Xinze Du. *Manuscript writing*: Xinze Du, Kexin Wang, Xiaodong Tian and Yongsu Ma. *Supervision*: Xiaoying Wang, Yinmo Yang, Xiaodong Tian and Yongsu Ma.

## CONFLICT OF INTEREST STATEMENT

The authors declare no conflicts of interest.

## ETHICAL APPROVAL

Ethical approval was obtained for this retrospective study [2019(170)]; the requirement to obtain informed consent was waived.

## Data Availability

All datasets, related codes, and data analysis scripts will be provided request to author Xinze Du (1610301126@pku.edu.cn).
